# Linaclotide activates guanylate cyclase‐C/cGMP/protein kinase‐II‐dependent trafficking of CFTR in the intestine

**DOI:** 10.14814/phy2.13299

**Published:** 2017-06-07

**Authors:** Md. Kaimul Ahsan, Boris Tchernychev, Marco M. Kessler, Robert M. Solinga, David Arthur, Cristina I. Linde, Inmaculada Silos‐Santiago, Gerhard Hannig, Nadia A. Ameen

**Affiliations:** ^1^ Department of Pediatrics/Gastroenterology and HepatologyYale School of MedicineNew HavenConnecticut; ^2^ Department of PharmacologyIronwood PharmaceuticalsCambridgeMassachusetts; ^3^ Biopta LtdGlasgowUnited Kingdom; ^4^ Department of Preclinical ResearchDecibel TherapeuticsBostonMassachusetts; ^5^ Department of Cellular and Molecular PhysiologyYale School of MedicineNew HavenConnecticut

**Keywords:** CFTR, chronic idiopathic constipation, irritable bowel syndrome with constipation, linaclotide, PKGII/PKA

## Abstract

The transmembrane receptor guanylyl cyclase‐C (GC‐C), expressed on enterocytes along the intestine, is the molecular target of the GC‐C agonist peptide linaclotide, an FDA‐approved drug for treatment of adult patients with Irritable Bowel Syndrome with Constipation and Chronic Idiopathic Constipation. Polarized human colonic intestinal cells (T84, CaCo‐2BBe) rat and human intestinal tissues were employed to examine cellular signaling and cystic fibrosis transmembrane conductance regulator (CFTR)‐trafficking pathways activated by linaclotide using confocal microscopy, in vivo surface biotinylation, and protein kinase‐II (PKG‐II) activity assays. Expression and activity of GC‐C/cGMP pathway components were determined by PCR, western blot, and cGMP assays. Fluid secretion as a marker of CFTR cell surface translocation was determined using in vivo rat intestinal loops. Linaclotide treatment (30 min) induced robust fluid secretion and translocation of CFTR from subapical compartments to the cell surface in rat intestinal loops. Similarly, linaclotide treatment (30 min) of T84 and CaCo‐2BBe cells increased cell surface CFTR levels. Linaclotide‐induced activation of the GC‐C/cGMP/PKGII signaling pathway resulted in elevated intracellular cGMP and pVASP ^ser239^ phosphorylation. Inhibition or silencing of PKGII significantly attenuated linaclotide‐induced CFTR trafficking to the apical membrane. Inhibition of protein kinase‐A (PKA) also attenuated linaclotide‐induced CFTR cell surface trafficking, implying cGMP‐dependent cross‐activation of PKA pathway. Together, these findings support linaclotide‐induced activation of the GC‐C/cGMP/PKG‐II/CFTR pathway as the major pathway of linaclotide‐mediated intestinal fluid secretion, and that linaclotide‐dependent CFTR activation and recruitment/trafficking of CFTR from subapical vesicles to the cell surface is an important step in this process.

## Introduction

Receptor guanylyl cyclase‐C (GC‐C) is a class I transmembrane receptor that belongs to a larger family of enzymes, including several particulate guanylyl cyclases and soluble guanylate cyclase (Lucas et al. 2000; Vaandrager 2002). GC‐C is expressed predominantly on epithelial cells throughout all segments of the gastrointestinal tract and plays an important role in intestinal electrolyte transport and fluid homeostasis (Swenson et al. 1996; Castro et al. 2013; Silos‐Santiago et al. 2013). The domain structure of GC‐C consists of an extracellular ligand‐binding domain, a single transmembrane domain, and the intracellular kinase homology and catalytic domains. Its intrinsic guanylyl cyclase activity is stimulated by binding of the natural hormones guanylin and uroguanylin resulting in conversion of guanosine triphosphate into the second messenger 3′,5′‐cyclic guanosine monophosphate (cGMP). Increased levels of intracellular cGMP are known to modulate a broad range of cellular processes through interaction with three groups of target proteins: cGMP‐dependent protein kinases, cGMP‐dependent phosphodiesterases, and cyclic nucleotide‐gated ion channels (Pfeifer et al. 1996; Schlossmann et al. 2005).

The synthetic GC‐C agonist peptide, linaclotide, is an FDA‐approved drug for adult patients with Irritable Bowel Syndrome with Constipation (IBS‐C) and Chronic Idiopathic Constipation (CIC) (Bryant et al. 2010; Busby et al. 2010). IBS‐C is a highly prevalent, chronic functional gastrointestinal disorder with a global prevalence estimated to range from 5% to 20% (Mayer 2008; Quigley et al. 2012). Symptoms of this disorder include recurrent abdominal pain or discomfort associated with a change in stool frequency or stool form that significantly impact patients' quality of life and results in high utilization of health care resources (Drossman 2006; Spiegel et al. 2009; Agarwal and Spiegel 2011). Studies using models of visceral pain provided evidence that the analgesic effects of linaclotide are mediated through a GC‐C‐dependent peripheral sensory mechanism, involving extracellular cGMP secreted in the submucosa, modulating intestinal nociceptor function (Castro et al. 2013; Silos‐Santiago et al. 2013). Chronic constipation is estimated to affect between 12% and 19% of the US population (Lembo and Camilleri 2003; Higgins and Johanson 2004). Symptoms of this functional bowel disorder include infrequent bowel movements, hard stools, bloating and abdominal discomfort, straining during defecation, and a sense of incomplete evacuation that adversely impact patient's quality of life and leads to a substantial economic burden on health care costs (Martin et al. 2006; Johanson and Kralstein 2007; Wald et al. 2007; Talley 2008). In models of gastrointestinal function, linaclotide‐mediated activation of GC‐C increased fluid secretion and accelerated intestinal transit, suggesting a key role for the GC‐C/cGMP pathway in the improvement of bowel symptoms (Bryant et al. 2010; Busby et al. 2010).

The cystic fibrosis transmembrane conductance regulator (CFTR) is a cyclic adenosine monophosphate (cAMP) and cGMP‐regulated anion‐selective channel that is expressed on the apical domain of intestinal epithelial cells where it serves as the main conductive pathway for Cl^−^ and HCO_3_^−^ ions transported across the membrane into the lumen (Riordan et al. 1989; Anderson et al. 1991; Vaandrager et al. 1997). Previous studies have shown increased transepithelial electrolyte secretion across the mucosa following linaclotide activation of the GC‐C/cGMP pathway, modulated by multidrug resistance‐associated protein 4 (MRP4) (Tchernychev et al. 2015). Moreover, studies investigating the physiological relevance of cell‐specific distribution patterns of CFTR and other ion transporters along the intestinal crypt–villus axis following in vivo cholinergic stimulation revealed CFTR trafficking and membrane recruitment from subapical vesicles (Jakab et al. 2011). This was further consistent with findings that in the unstimulated intestine, CFTR is partially localized to subapical intracellular vesicles, indicative of regulation by recycling (Jakab et al. 2011). Finally, in addition to regulation by subcellular trafficking, CFTR ion channel activity is further regulated by protein kinase‐A (PKA) and cGMP‐dependent protein kinase‐II (PKGII or cGKII) phosphorylation of its intracellular regulatory domain. The effect of linaclotide on CFTR regulation and function, particularly the effect of linaclotide‐dependent GC‐C/cGMP pathway activation on subcellular CFTR trafficking patterns has not yet been reported.

In this study, we have investigated cellular events following linaclotide‐induced activation of the GC‐C/cGMP pathway using polarized in vitro intestinal epithelial cell models and in vivo assays of rat intestine and human colonic tissues. Furthermore, we provide evidence for the involvement of the cGMP/PKGII pathway and possible involvement of the PKA pathway in linaclotide‐stimulated cell surface trafficking of CFTR, a critical step in the regulation of intestinal electrolyte and fluid homeostasis.

## Materials and Methods

### Reagents and antibodies

Linaclotide was provided by Ironwood Pharmaceuticals Inc (Cambridge, MA). EZ‐Link Sulfo‐NHS‐SS‐Biotin and streptavidin‐agarose resins were obtained from Thermo Scientific (Rockford, IL). 8‐Bromoguanosine 3′,5′‐cyclic monophosphate sodium salt monohydrate (8Br‐cGMP), bumetanide, GlyH‐101, and NKH477 were obtained from Sigma‐Aldrich (St. Louis, MO). The PKA inhibitor H7 and PKG inhibitor H8 dihydrochloride were purchased from AbCam (Cambridge, MA). The mouse monoclonal CFTR‐450 antibody raised against full‐length human CFTR was obtained from Cystic Fibrosis Foundation Therapeutics, University of North Carolina‐Chapel Hill. The affinity‐purified polyclonal antibody AME4991 raised against rat CFTR was generated by Dr. Ameen (Ameen et al. 2000; Golin‐Bisello et al. 2005; Jakab et al. 2011). Phospho‐VASP (Ser239) and antimouse IgG, HRP‐linked antibodies were obtained from Cell Signaling Technology Inc. (Danvers, MA). The cGKII (PRKG2) rabbit polyclonal antibody was obtained from Proteintech Group Inc. (Chicago, IL) and β‐actin monoclonal antibody from Sigma‐Aldrich (St. Louis, MO). All other drugs, reagents, and chemicals were obtained from Sigma‐Aldrich unless otherwise stated.

### Animals

All animal studies were approved by the Institutional Animal Care and Use Committee of Yale University School of Medicine. Briefly, male Sprague–Dawley rats (200–250 g, Charles River Laboratories) were fasted overnight with access to drinking water ad libitum. Anesthesia was performed with Inactin^®^ (Thiobutabarbital sodium salt hydrate, 120 mg/kg/i.p.). The jejunal, ileal, and proximal colonic loops (5 cm length) were prepared using sutures. Loops were instilled either with normal saline (pH7.4, 37°C) (1 mL/loop) or linaclotide (5 μg/mL/loop) for 30 min. At the end of the study, animals were euthanized with Inactin (200 mg/kg/i.p.).

### Analysis of Transepithelial Short Circuit Current (I_sc_) in rat and human colonic mucosa

Recordings of transepithelial ion current across rat colonic mucosa were conducted as described previously (Silos‐Santiago et al. 2013; Tchernychev et al. 2015). Briefly, proximal colons were collected from Sprague–Dawley rats (n = 4–6/per group) and seromuscular layers were removed by blunt dissection. Linaclotide was added to the apical bath of the Ussing chamber at indicated concentrations. Where indicated, the CFTR inhibitor GlyH‐101 (0.1 mmol/L) was added to the apical bath 20 min after linaclotide stimulation. To study the effect of PKGII inhibition on linaclotide‐induced I_sc_ colonic epithelium was preincubated for 30 min with PKGII inhibitor H8 (0.1 mmol/L) before stimulation with linaclotide.

Ascending colon samples were obtained by full informed consent and ethical permission from patients undergoing surgical procedures (ReproCELL USA Inc, Boston MA). Tissue sections were then mounted on Ussing sliders with a 0.5 cm^2^ aperture. A total volume of 5 mL of Physiological Saline Solution (PSS) containing 119 mmol/L NaCl, 4.7 mmol/L KCl, 1.2 mmol/L MgSO_4_, 24.9 mmol/L NaHCO_3_, 1.2 mmol/L KH_2_PO_4_ and 2.5 mmol/L CaCl_2_ at pH 7.4 was added to the apical and basolateral chambers of the Ussing chamber. The apical and basolateral chambers also contained mannitol (11.1 mmol/L) and glucose (11.1 mmol/L), respectively. Both baths were perfused with 95% O_2_ and 5% CO_2_ and maintained at a constant temperature of 37°C throughout the duration of the experiment. Linaclotide was added to the apical bath of Ussing chamber and Na^+^/K^+^/Cl^−^ cotransporter 1 (NKCC1) inhibitor bumetanide (100 μmol/L) was added to the serosal bath to inhibit the I_sc_ associated with Cl^−^ ion transport. Data are presented as ΔI_sc_ and ΔI_sc_ (% of max). Changes in I_sc_ values (ΔI_sc_) were calculated using the equation $\Delta I_{\mathrm{sc}}=-\left(\right|I_{\mathrm{sc}}\left|-\right|I_{{\mathrm{sc}}^{\min}}\left|\right)$ and GraphPad Prizm Software (GraphPad Software, San Diego, CA), where |I_sc_| represents the absolute value of I_sc_ recorded at a given time, and $|I_{{\mathrm{sc}}^{\min}}|$ represents a minimal absolute value of I_sc_ recorded at baseline.

### Cell cultures

Human colon cell lines CaCo‐2BBe (hereafter referred as C2BBe) and T84 cells were obtained from ATCC (Manassas, VA). C2BBe cells were grown in high glucose (4.5 g/L) DMEM (Gibco), supplemented with 10% FBS (Gibco), 10 μg/mL apo‐transferrin (Sigma‐Aldrich), 1 mmol/L sodium pyruvate (Gibco), and 1× Anti‐Anti (Gibco). T84 cells were grown in DMEM/F‐12 medium, supplemented with 5% FBS (Gibco), 1× Anti‐Anti (Gibco) in a 37°C incubator with 5% CO_2_–90% air atmosphere. At 70% confluence, cells were passaged onto 0.4‐μm polycarbonate membrane 24‐mm Transwell filters (Costar Inc.) at 1 × 10^5^ cells/cm^2^ density. After 3–4 weeks, filter‐grown, fully mature, polarized, and confluent monolayer cells were used for surface biotinylation experiments and Western blot analyses.

### Immunofluorescence labeling and Confocal Microscopy

After completion of treatment either with normal saline or linaclotide for 30 min, intestinal segments were immediately removed and briefly rinsed with ice‐cold PBS. Tissues were cut into ~2‐mm‐thick rings, fixed in 2% paraformaldehyde in PBS, pH 7.4 for 1 h at room temperature, rinsed with PBS and cryoprotected in 30% sucrose overnight. Multiple tissue samples were processed under identical conditions by the utilization of tissue arrays as previously described (Jakab et al. 2011). Frozen sections were cut (5 μm in thick) on a cryostat, mounted on Superfrost^®^ Plus slides (Fisher Scientific), and immunolabled as described (Jakab et al. 2012). Immunolabeled sections were stored at 4°C and examined on Zeiss LSM 710 DUO laser scanning confocal microscope outfitted with a Mai‐Tai laser and electronics capable of spectrally separating fluorescence emission profiles. Image acquisition and processing were performed using ZEN (Zeiss Efficient Navigation) software. The acquisition parameters were standardized in relation to the highest‐intensity regions to avoid oversaturation of pixel intensity.

### Surface biotinylation

Surface biotinylation of cell lines was performed as previously described (Silvis et al. 2009; Collaco et al. 2010) after treatment either with 8Br‐cGMP or linaclotide in the presence or absence of H7 (PKA inhibitor) or H8 (PKG inhibitor). Briefly, cells were washed twice with ice‐cold PBS containing 0.1 mmol/L CaCl_2_ and 1.0 mmol/L MgCl_2_ (PBS‐CM) and immediately placed on ice in a cold room. Rat intestine surface biotinylation was performed using a modified protocol as previously described (Ameen et al. 2003; Golin‐Bisello et al. 2005; Ameen and Apodaca 2007), immediately after treatment either with normal saline (pH7.4, 37°C) or linaclotide (5 μg/mL/loop) in vivo. Intestinal loops were incubated with freshly prepared Sulfo‐NHS‐SS‐biotin (1 mg/mL) in ice‐cold PBS‐CM (pH 8.0) in the cold room and processed as described (Golin‐Bisello et al. 2005). Cell surface and intestinal tissue biotinylated proteins were dissociated from streptavidin agarose by 2× SDS sample buffer (BioRad). Cell lysates (30 μg) and biotinylated samples were separated by SDS‐PAGE to detect CFTR by Western Blot analysis.

### Western blots

C2BBe or T84 cells were homogenized in TGH lysis buffer [25 mmol/L HEPES, 10% (vol/vol) glycerol, 1% (vol/vol) Triton‐ × 100, pH 7.4] containing protease inhibitors (10 nmol/L iodoacetamide, 1 mmol/L phenylmethylsulfonyl fluoride (PMSF), 2 μg/mL leupeptin) for 30 min on ice. Homogenates were solubilized, subject to centrifugation at 13,200 *g* for 15 min at 4°C, and cleared supernatants were recovered. Protein concentration of the supernatant was determined with Coomassie Protein Assay Reagent (Pierce) and samples prepared and analyzed as described (Kravtsov et al. 2016). Quantification of surface‐labeled proteins was performed with Quantity One image analysis software.

### Semiquantitative RT‐PCR

For the mRNA expression analyses of cGKII, total RNA was isolated from C2BBe and T84 cells using Trizol reagent (Invitrogen). cDNA was synthesized using a SuperScript First‐Strand Synthesis System (Invitrogen) with oligo dT_12–18_ according to manufacturer instructions. The cDNA was amplified by PCR using a Taq DNA polymerase (Qiagen). The primers used for the amplification were as follows: cGKII, 5′‐GGTCCCTGAGCAAAATGGGA‐3′ (forward) and 5′‐GCTTTCACAGAGGCAGTCCT‐3′ (reverse); and GAPDH, 5′‐ATGGGGAAGGTGAAGGTCGGAGTC‐3 (forward) and 5′‐CCATGCCAGTGAGCTTCCCGTTC‐3′ (reverse). PCR conditions were as follows: 35 cycles for cGKII (denaturing at 94°C for 30 sec, annealing at 56°C for 1 min, and extension at 72°C for 1:30 min), and 22 cycles for GAPDH (denaturing at 94°C for 30 sec, annealing at 61°C for 1 min, and extension at 72°C for 1:30). The PCR products were visualized by electrophoresis in 2% agarose gel (Sigma‐Aldrich) containing 1 μg/mL ethidium bromide.

### Measurement of cGMP in human tissues and supernatants

Nontransplantable intestinal tissue samples from the jejunum, ileum, and ascending colon were obtained with proper authorization and full ethical approval from four organ donors with no known history of gastrointestinal disease (ReproCELL, Boston MA). Tissue samples were washed with physiological saline solution (PSS composition: 119.0 mmol/L NaCl, 4.7 mmol/L KCl, 1.2 mmol/L MgSO_4_, 24.9 mmol/L NaHCO_3_, 1.2 mmol/L KH_2_PO_4_, 2.5 mmol/L CaCl_2_, and 11.1 mmol/L glucose). Each of the three intestinal segments was dissected in half and mucosa was isolated from each segment. All mucosal segments were placed in PSS containing protease inhibitors. The mucosa was further dissected into small sections of approximately 100 mg. The mucosa sections were transferred to individual tubes with prewarmed PSS (37°C) containing 1 mmol/L of the phosphodiesterase inhibitor 3‐isobutyl‐1‐methylxanthine (IBMX). Samples were incubated at 37°C for indicated times either in 0.2‐mL sterile reverse osmosis (RO) water or a solution containing 1 μmol/L linaclotide. Water was chosen as formulation in order to assess effect of linaclotide on the levels of extracellular cGMP. Following incubation, the mucosa samples were rinsed with cold PBS and quickly blot dried. Supernatants and the mucosa sections were transferred into separate vials and flash frozen in liquid nitrogen. Frozen samples (100 mg tissue) were transferred to an ice bath and 0.3 mL of ice‐cold 6% trichloroacetic acid was added to the samples. The mucosa was homogenized in an Omni Homogenizer (Model TH with a 7 mm × 190 mm saw tooth stainless steel probe) at maximum speed for 5 sec at 4°C. The homogenate was then centrifuged at 16,000 *g* at 4°C and the total protein concentration in the cleared homogenate was measured using a Bradford assay. The concentration of cGMP in tissue homogenates and supernatants was determined using a cGMP Enzyme immunoassay Biotrak (EIA) System (GE Healthcare Amersham). The concentration of cGMP in tissues was expressed as pmol/mg protein and as nmol/L concentration in supernatants.

To measure cGMP in supernatants of linaclotide‐stimulated human colonic cell lines, T84 and C2BBe cells were plated into 96‐well plate (200,000 cells/well) 48 h prior to the study. On the day of the study cells were washed and incubated for 10 min at 37°C with 1 mmol/L IBMX in 0.18 mL of Dulbecco's modified Eagle's medium (DMEM; pH 7). At the end of incubation 0.02‐ml aliquots of linaclotide (concentration range: 0.001 μmol/L–10 μmol/L) was added in duplicate to cells. The plates were then incubated for 30 min at 37°C and supernatants were collected for cGMP measurements. Concentrations of cGMP in supernatants of T84 and C2BBe cells were determined using liquid chromatography with tandem mass spectroscopy (LC/MS/MS).

### Knockdown of cGKII expression in C2BBe and T84 cells

cGKII mRNA was targeted with shRNA delivered by a lentiviral system based on a pLKO.1‐Puro vector. Cells were transduced to stably express scrambled or cGKII shRNA. cGKII‐targeting shRNA (5′‐GGGCCTTAATAACCATTTAGT‐3′) was designed with the Bioinformatics & Research Computing online tool **(** http://jura.wi.mit.edu/bioc/siRNAext/ **)** and subcloned into *Age*I and *Eco*RI restriction sites of pLKO.1‐TRC vector (Addgene no. 10878), immediately downstream from the U6 promoter. The pLKO.1‐Puro vector containing the scrambled shRNA was obtained from Addgene (no. 1864). The Lenti‐X™ 293T (HEK 293T; Clontech) packaging cell line was transfected with these shRNA constructs using X‐tremeGENE 9 DNA transfection reagent (Roche Diagnostics, Indianapolis, IN) according to the Addgene's protocol. Lenti‐X™ 293T cells (7 × 10^5^) were plated in 6‐cm well plate and transfected the following day at 60–70% confluence with 1 μg of shRNA containing vector, 0.75 μg of packaging plasmid (psPAX2, Addgene no. 12260), and 0.25 μg of envelope plasmid (pMD2.G, Addgene no. 12259). After 15 h incubation at 37°C, the medium was aspirated and replaced with fresh medium. Virus‐containing medium was collected at 24 h and 48 h after transfection and titrated for optimal multiplicity of infection (MOI). C2BBe cells seeded at 4 × 10^5^/mL were transduced at 60–70% confluence with Polybrene (Millipore) at 8 μg/ml final concentration. After transduction, cells were selected with 12 μg/mL puromycin‐containing medium (Sigma‐Aldrich). Efficiency of shRNA silencing was determined by Western blot. Surface biotinylation was performed on cells as described above.

### Statistical analysis

Data are expressed as the mean ± SEM or SD. The significance of differences in mean values was determined using the two‐tailed Student's *t*‐test. *P* values < 0.05 are considered significant.

## Results

### Linaclotide stimulates fluid secretion by recruiting CFTR to the apical surface of rat small intestine

**Figure 1:**
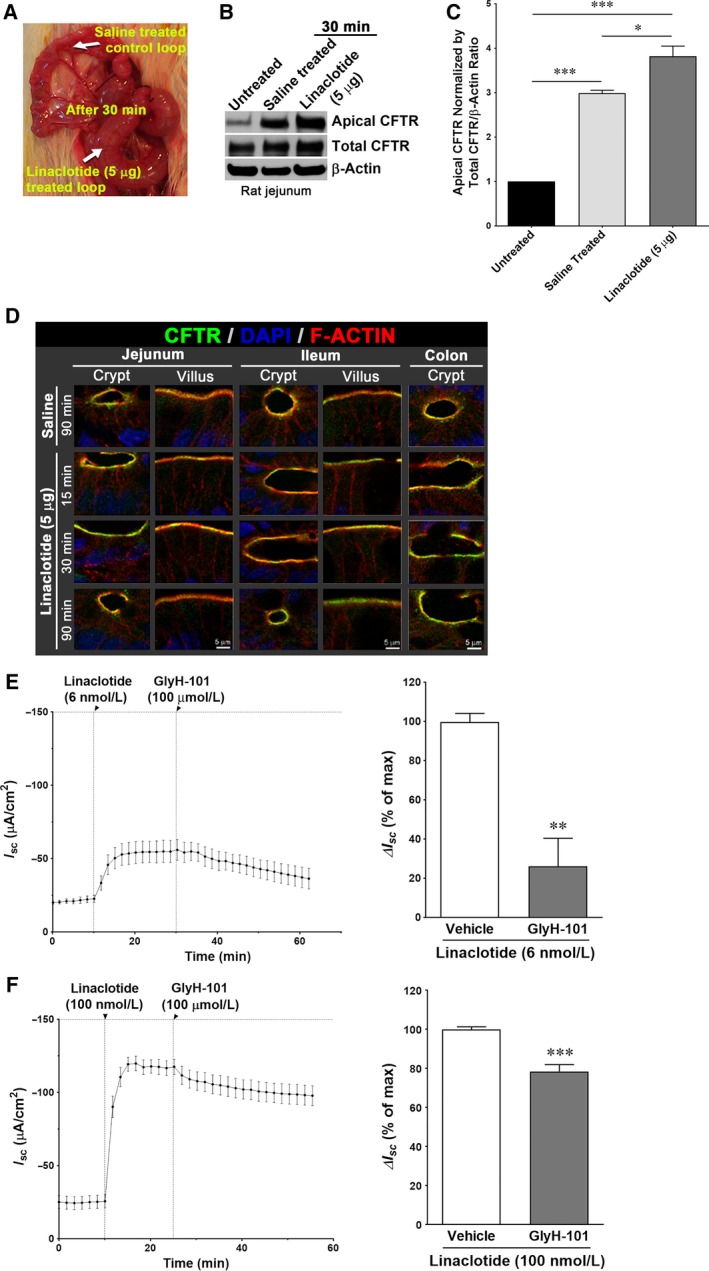
Linaclotide‐induced apical trafficking/recruitment of CFTR and transepithelial CFTR ion current in rat intestine. Rat intestinal loops from different segments were treated with saline or linaclotide (5 μg/mL/loop) for 30 min and surface CFTR was analyzed using biochemical assays and immunofluorescence labeling. Normal saline was used as a vehicle treatment. (A) Gross appearance of rat jejunal loops after 30 min treatment with linaclotide (5 μg) or normal saline. (B) Following treatment with linaclotide (5 μg) or saline, rat jejunal enterocytes were collected and surface biotinylated proteins were analyzed to detect CFTR and analyzed by Western blot. β‐actin was used as a loading control. (C) Representative bar graph shows normalized surface CFTR relative to saline‐treated and untreated jejunum. Data are presented as Mean ± SEM (*n* = 4). (D) Confocal microscopic images of CFTR (green) in relation to F‐actin (red), nuclei are stained in blue; IFL in the crypts and villi of various segments of rat intestine treated with or without linaclotide (5 μg) for 30 min. Scale bar, 5 μm. Inhibition of CFTR attenuates linaclotide‐induced transepithelial ion current in rat ascending colon stimulated with linaclotide 6 nmol/L (E) and 100 nmol/L (F). After the short circuit plateau was reached, CFTR inhibitor GlyH‐101 was added to the apical bath. Transepithelial ion current (I_sc_) was recorded and the percent of inhibition by GlyH‐101 was determined for vehicle and GlyH‐101‐treated tissues. Representative recordings of short circuit current traces before and after the treatment of linaclotide (6 nmol/L; E, *left panel* and 100 nmol/L; F, *left panel*) followed by treatment of GlyH‐101 (100 μmol/L) in the rat ascending colon. Data are presented as mean ± SEM (*n* = 4).

The guanylate cyclase‐C (GC‐C) agonist linaclotide stimulates the production and accumulation of intracellular cGMP, which acts as a second messenger to enhance fluid secretion in the intestine (Schulz et al. 1990; Busby et al. 2013). We first tested whether linaclotide can enhance fluid secretion in vivo in the rat small intestine. Following anesthesia and laparotomy, intestinal loops were created in rat jejunum and injected either with vehicle (saline, 1 mL) or linaclotide (5 μg/1 mL). Following a 30‐min treatment period, the linaclotide‐treated loop appeared distended and tightly filled with fluid (Fig. 1A), compared to saline, indicating enhanced fluid secretion over the 30‐min period. Our previous study indicated that cGMP‐mediated fluid secretion is mediated by CFTR ion channel translocation to the apical surface in rat jejunum (Golin‐Bisello et al. 2005). In this study, we examined whether linaclotide treatment also stimulates fluid secretion by recruiting CFTR to the apical surface of rat jejunum enterocytes in vivo. Surface biotinylation was performed on untreated rat enterocytes, or 30 min following linaclotide (5 μg) treatment of rat jejunum loops in vivo. Western blot analysis of surface biotinylated proteins revealed an increase in surface CFTR in saline‐ and linaclotide‐treated samples with highest levels of surface CFTR in linaclotide‐treated enterocytes (Fig. 1B, C). High‐magnification confocal images of CFTR/F‐Actin immunolabeled cryosections from rat intestine were consistent with the results using surface biotinylation (Fig. 1D), indicating linaclotide‐induced accumulation of CFTR in the brush border (Fig. 1B–D).

We have previously shown that in the rat proximal colon, linaclotide potently increases transepithelial ion current (EC_50_ = 9.2 nmol/L) (Tchernychev et al. 2015). To further characterize the CFTR‐mediated electrogenic anion current, rat proximal colonic mucosa was stimulated with linaclotide at a concentration below the EC_50_ (6 nmol/L; Fig. 1E) or a saturated concentration of linaclotide (100 nmol/L; Fig. 1F), in the presence or absence of CFTR inhibitor GlyH‐101. Linaclotide induced a transepithelial ion current at both concentrations, while GlyH‐101 significantly attenuated the linaclotide‐evoked I_sc_, confirming CFTR activation and its contribution to transmucosal ion flux (Fig. 1E, F). Its inhibitory effect was more profound when tissues were stimulated with lower concentrations of linaclotide.

### Cyclic GMP‐dependent kinase cGKII is expressed in C2BBe and T84 cells

**Figure 2:**
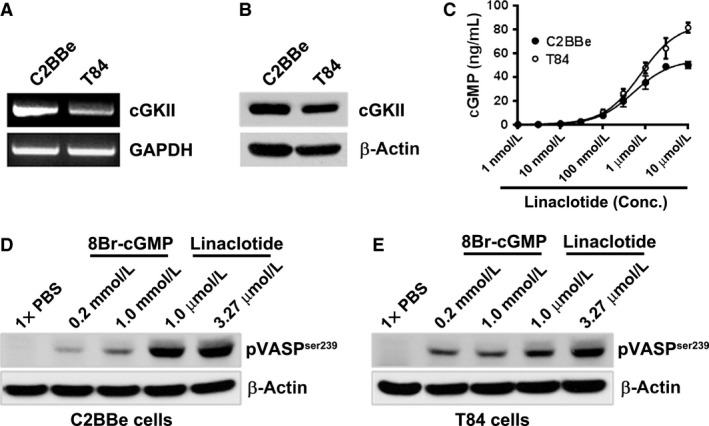
Expression levels of cGKII mRNA and protein, linaclotide‐stimulated production of cGMP and phosphorylation of VASP at serine 239 in the C2BBe and T84 cells. (A) Expression of cGKII mRNA in the C2BBe and T84 cells. (B) Protein levels of cGKII in C2BBe and T84 cells. (*C*) Linaclotide induces cGMP synthesis in C2BBe and T84 cells in a concentration‐dependent manner. Confluent cultures were pretreated with IBMX (1 mmol/L) for 10 min followed by stimulation with linaclotide (0.001 μmol/L–10 μmol/L) for 30 min. Concentration of cGMP in supernatants was determined using LC/MS/MS. Data are presented as Mean ± SD (*n* = 4). Western blot analysis of the phosphorylation status of VASP at Ser239 using two different doses of 8Br‐cGMP (0.2 mmol/L and 1 mmol/L) and linaclotide (1 μmol/L and 3.27 μmol/L) in the C2BBe (D) and T84 cells (E)**.** β‐actin served as a loading control.

The observation that linaclotide stimulated fluid secretion and CFTR trafficking into the brush border surface in the rat small intestine in vivo led us to investigate the mechanisms of linaclotide‐mediated downstream signaling pathways involved in fluid secretion. cGKII is essential for eliciting cGMP‐mediated fluid secretion in the intestine. cGKII knockout mice fail to elicit intestinal fluid secretion when treated with heat‐stable *E. coli* STa enterotoxin. STa is known to employ the cGMP/cGKII pathway to elicit excessive fluid secretion (Pfeifer et al. 1996). Previous studies in rat intestine indicated that cGKII expression is most abundant in villus enterocytes of the jejunum with lower levels in the proximal colon but no expression is detected in distal colon (Markert et al. 1995). We used the T84 cells, a crypt‐like intestinal cell model and C2BBe cells, a villus resembling intestinal cell model to examine linaclotide‐induced signaling pathways. Both cell models were derived from human *colorectal carcinoma cells* (Murakami and Masui 1980; Peterson and Mooseker 1992). Although T84 cells express cGKII mRNA (Selvaraj et al. 2000), expression of cGKII in C2BBe cells has not been reported. To confirm that the essential signaling machinery is present in both C2BBe and T84 cells, we first examined cGKII mRNA and protein expression in both intestinal epithelial cell lines (Fig. 2A, B). We found that cGKII mRNA and protein levels were higher in the villus resembling C2BBe cells, compared to crypt‐like T84 cells.

Previous reports indicated that T84 cells express GC‐C which can be activated by the natural hormones guanylin and uroguanylin and the heat stable enterotoxin STa to increase production of cGMP (Forte et al. 1993). Linaclotide concentration dependently stimulated cGMP synthesis in T84 and C2BBe cells (Fig. 2C). The potency of linaclotide in T84 cells was similar to that in C2BBe cells, with EC_50_ values of 772 nmol/L and 523 nmol/L, respectively.

### Linaclotide increases phosphorylation of VASP (vasodilator‐stimulated phosphoprotein) at Serine 239 in the C2BBe and T84 cells

VASP was originally characterized as a substrate for both PKG (cGMP‐dependent kinase) and PKA (cAMP‐dependent kinase). VASP possesses three phosphorylation sites, Ser157, Ser239, and Thr278 (Butt et al. 1994; Smolenski et al. 1998), and VASP phosphorylation at Ser239 has previously been reported to measure the activation status of cGKII (PKGII) (Oelze et al. 2000; Lawrence and Pryzwansky 2001). To determine linaclotide‐induced activation of cGKII, we determined the phosphorylation of VASP^Ser239^ 30 min following treatment either with vehicle or linaclotide (1 μmol/L and 3.27 μmol/L; the 3.27 μmol/L concentration is equivalent to the 5 μg dose of linaclotide that was used in vivo in the rat jejunum loop). The membrane permeant cGMP analog, 8 Br‐cGMP (0.2 mmol/L, 1 mmol/L), was used as a positive control. Linaclotide induced accumulation of cGMP in both human colonic cell lines in a concentration‐dependent manner (Fig. 2C) and importantly, more strongly enhanced phosphorylation of VASP^Ser239^ compared to the cGMP analog 8‐Br‐cGMP, in both C2BBe (Fig. 2D) and T84 cells (Fig. 2E). These data confirm that linaclotide activates cGKII by phosphorylation in its downstream signaling pathway.

### Linaclotide stimulates cGMP accumulation and short circuit current in human intestinal mucosa

**Figure 3:**
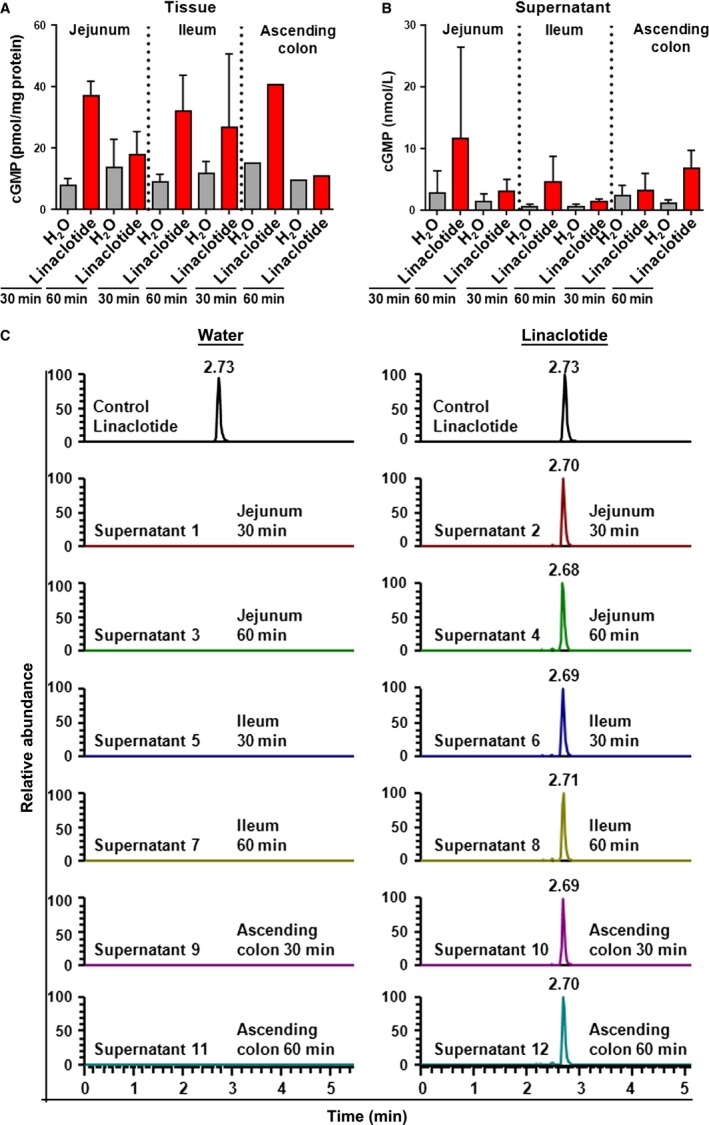
Linaclotide stimulates cGMP accumulation in human mucosa tissues and supernatants**.** Samples of human intestinal mucosa from the duodenum, ileum, and ascending colon were incubated in the presence of 1 μmol/L linaclotide or in water for 30 or 60 min at 37°C. After the incubation, tissues were homogenized and cGMP was extracted from the homogenized tissues in TCA. Cyclic GMP was measured in clear homogenates (A) and in reaction supernatants (B) using a competitive enzyme immunoassay. Presence of intact linaclotide in tissue supernatants after incubation was confirmed by LC/MS. C. Profile of extracted ion reverse‐phase HPLC chromatograms of linaclotide after detection with mass spectrometry. The presence of linaclotide is shown by the chromatographic peak with a retention time of 2.7 min. The *y*‐axis shows relative abundance in %. All data are expressed as mean ± SD (*n* = 4).

To measure GC‐C activity in biopsies of human jejunum, ileum, and ascending colon, we measured levels of cGMP generated in tissue samples following GC‐C activation by linaclotide. Biopsies were incubated in a bath containing 1 μmol/L linaclotide, and cGMP levels were measured by ELISA in tissues (intracellular) and medium (extracellular/supernatant). Figure 3 shows the increase in cGMP levels in tissue (Fig. 3A) and supernatants (Fig. 3B) following stimulation with linaclotide for 30 and 60 min. The increase in cGMP levels was greater after 30 min, compared to levels measured after 60 min. In parallel, the stability of linaclotide in tissue supernatants after the 30 and 60 min incubation periods was analyzed by LC‐MS, which confirmed that linaclotide is stable under these conditions (Fig. 3C).

**Figure 4:**
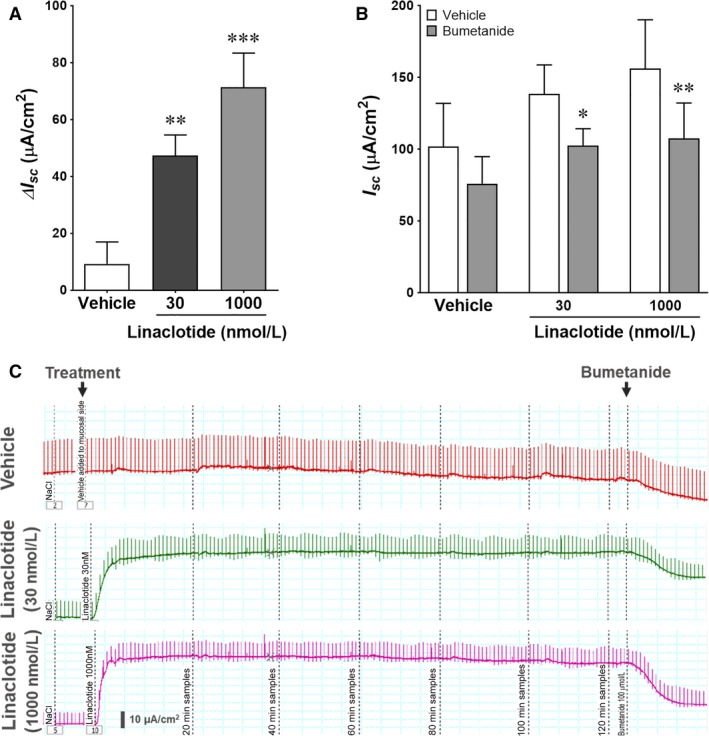
Linaclotide induces CFTR ion current in the human colonic mucosa. (A) Linaclotide induces transepithelial ion current across human colonic mucosa. Intestinal epithelium isolated from human ascending colon was stimulated with either vehicle (*water*) or linaclotide (30 nmol/L and 1000 nmol/L). Transepithelial CFTR ion current (Isc) was recorded and change in transepithelial ion current (ΔIsc) was determined as the difference between basal Isc and maximal Isc response after application of vehicle or linaclotide. Data are presented as mean ± SEM (*n* = 12). (B) Effect of bumetanide on linaclotide‐induced transepithelial ion current. Inhibition of NKCC1 with bumetanide inhibits GC‐C mediated short circuit current across human colonic mucosa. Intestinal epithelium isolated from human ascending colon was stimulated with either vehicle (water) or linaclotide (30 nmol/L and 1000 nmol/L). NKCC1 inhibitor bumetanide (100 μmol/L) was added to basolateral bath and recording was continued. Data are presented as mean ± SEM (*n* = 6) of absolute Isc values before and after bumetanide treatment. (C) Representative recordings of short circuit current before and after treatment of human colonic epithelium with vehicle, linaclotide, and bumetanide. Epithelial tissues used for these recordings were dissected from the same ascending colon.

Both natural GC‐C agonists guanylin and STa increase electrogenic Cl^−^ secretion in different segments of human intestine including the ascending colon (Kuhn et al. 1994). To evaluate the effect of linaclotide on electrolyte secretion in human intestine, human ascending colonic mucosa was stimulated either with vehicle or linaclotide. Treatment of human colonic enterocytes with linaclotide induced transepithelial ion current at concentrations of 30 nmol/L and 1000 nmol/L (Fig. 4A, C). To address the question whether this current is mediated by transepithelial flux of Cl^−^ anions, we used NKCC1 inhibitor bumetanide. Addition of bumetanide to the basolateral chamber significantly attenuated linaclotide‐evoked I_sc_ (Fig. 4B, C).

### Linaclotide induces CFTR recruitment to the apical surface of C2BBe and T84 cells

**Figure 5:**
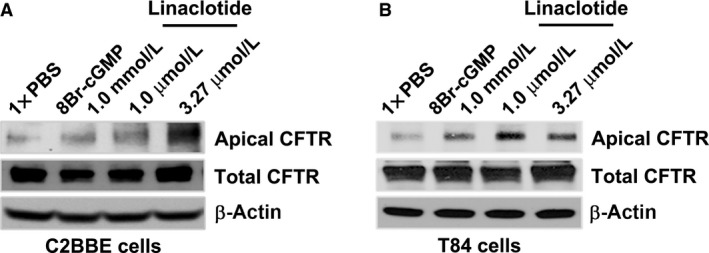
Linaclotide induces trafficking of CFTR to the cell surface of C2BBe and T84 cells. Filter‐grown fully mature, polarized cell monolayers were treated with a single concentration of 8Br‐cGMP (1 mmol/L; positive control) and two concentrations of linaclotide (1 μmol/L, 3.27 μmol/L) for 30 min. Surface biotinylated labeled cells were lysed and biotinylated proteins were recovered by streptavidin‐agarose coprecipitation and analyzed by Western Blot to detect CFTR in C2BBe cells (A) and T84 cells (B). β‐actin was used as a loading control.

Next, we examined whether the linaclotide‐induced activation of cGKII modulates CFTR traffic to the cell surface of C2BBe and T84 cells. First, we examined the phosphorylation status of VASP^Ser239^ as it is a marker of cGKII activity (Oelze et al. 2000; Lawrence and Pryzwansky 2001). Surface biotinylation was performed 30 min after treatment with linaclotide (1 μmol/L, 3.27 μmol/L) or PBS, pH 7.4. As before, the cGMP agonist, 8 Br‐cGMP (1 mmol/L), served as a positive control. Treatment of cells with linaclotide resulted in robust recruitment of CFTR to the apical surface of C2BBe (Fig. 5A) and T84 cells (Fig. 5B) in a concentration‐dependent manner, correlating with linaclotide‐induced phosphorylation of VASP^Ser239^ (Fig. 2D, E). These data indicate that linaclotide‐induced traffic of CFTR to the cell surface depends on activation and phosphorylation of cGKII/PKGII.

### Knockdown of cGKII inhibits linaclotide‐induced CFTR recruitment to the apical surface of C2BBe cells

**Figure 6:**
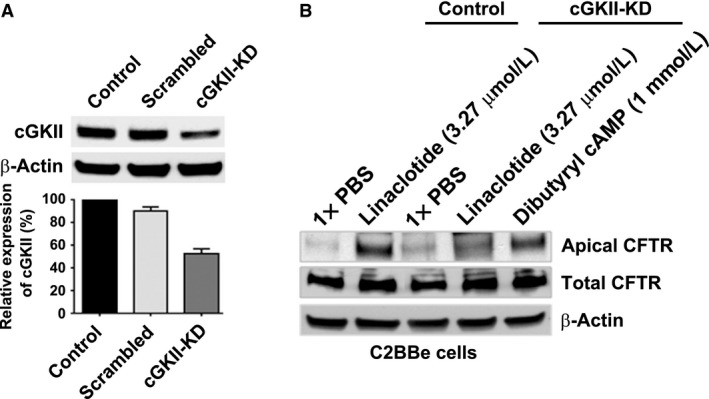
Knockdown of cGKII inhibits linaclotide‐induced but not dibutyryl cAMP‐induced apical cell surface trafficking of CFTR in the C2BBe cells. (A) cGKII knockdown was performed using lentiviral cGKII shRNA transduction and knockdown efficacy was analyzed by Western blots. (B) Filter‐grown fully mature, polarized monolayers of wild‐type, scrambled, and lentiviral cGKII shRNA‐transduced cells were treated with a single concentration of linaclotide (3.27 μmol/L) or dibutyryl cAMP (1 mmol/L) for 30 min. Surface proteins were labeled with biotin, the cells were lysed, biotinylated proteins were recovered by streptavidin‐agarose coprecipitation and analyzed by Western blot to detect CFTR. Total CFTR in C2BBe cell lysates was analyzed by Western blot. β‐actin served as a loading control.

To independently confirm that cGKII is necessary for linaclotide‐induced CFTR traffic to the cell surface, we silenced (knockdown) the expression of cGKII in the C2BBe cells and examined CFTR surface expression by surface biotinylation in presence or absence of linaclotide treatment for 30 min. Transduction of lentiviral cGKII shRNA was used to knockdown cGKII in the C2BBe cells, with downregulation of approximately 50 ± 3% (Fig. 6A). Surface CFTR levels were examined in cGKII knockdown (cGKII‐KD) in C2BBe cells following treatment with (a) PBS, (b) linaclotide (3.27 μmol/L), or (c) an analog of cAMP, Dibutyrl cAMP (1 mmol/L), as PKA can also stimulate CFTR traffic into the cell surface. Surface CFTR was reduced following linaclotide treatment when compared to PBS‐treated cells, while Dibutyryl cAMP treatment increased apical surface CFTR (Fig. 6B). These data confirm that the cGMP‐cGKII signaling pathway is important in mediating linaclotide‐induced traffic of CFTR and fluid secretion into the intestinal lumen.

### Inhibition of PKA and PKG abrogate linaclotide‐induced recruitment of CFTR to the apical surface of C2BBe and T84 cells

**Figure 7:**
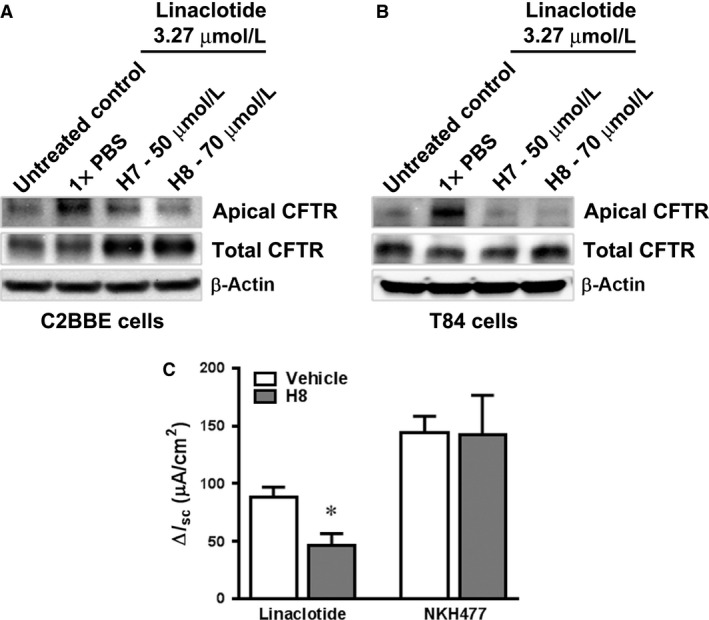
The PKA inhibitor H7 and PKG inhibitor H8 both reduce linaclotide‐induced apical trafficking of CFTR in the C2BBe and T84 cells. Filter‐grown fully mature, polarized cell monolayers were treated with a single concentration of linaclotide (3.27 μmol/L) for 30 min in the presence or absence of pretreatment with H7 (50 μmol/L) or H8 (70 μmol/L) for 30 min. Surface proteins were labeled with ice‐cold sulfo‐NHS‐SS‐biotin (1 mg/ml), cells were lysed, biotinylated proteins were recovered by streptavidin‐agarose coprecipitation, and analyzed by Western blot to detect CFTR in C2BBe cells (A) and T84 cells (B). β‐actin served as a loading control. (C) Effect of PKGII inhibition on linaclotide‐induced transepithelial current. Inhibition of PKGII significantly attenuates GC‐C but not adenylyl cyclase‐mediated transepithelial ion current. Intestinal epithelium isolated from rat proximal colon was mounted into Ussing chamber and incubated for 30 min with the PKGII inhibitor H8 (100 μmol/L) or vehicle (DMSO). GC‐C and adenylyl cyclase was activated by linaclotide (100 nmol/L) and NKH477 (20 μmol/L) added to apical and basolateral bath correspondingly. Change in transepithelial ion current (ΔI_sc_) was determined as the difference between basal I_sc_ and maximal I_sc_ response after application of linaclotide or NKH477. Data are presented as the mean ± SEM (*n* = 5).

Our previous studies examining the effects of STa enterotoxin in rat jejunum employed pharmacologic inhibitors of PKA (H7) and PKGII (H8) and similar approaches as utilized in this study to demonstrate that STa‐stimulated CFTR traffic to the cell surface was PKG but not PKA dependent (Golin‐Bisello et al. 2005). In addition, earlier studies also suggested that in the colon, STa activation of fluid secretion occurs through cGMP cross‐activation of PKA to regulate CFTR traffic, as cGKII is absent in this intestinal segment (Vaandrager et al. 1997). Because both C2BBe and T84 cells are derived from the colon, we examined both PKG and PKA signaling pathways in these cells. Using the surface biotinylation approach, we examined surface CFTR in the presence or absence of H7 or H8 in both C2BBe cells (Fig. 7A) and T84 cells (Fig. 7B). As predicted, linaclotide (3.27 μmol/L) treatment for 30 min induced a robust increase in surface levels of CFTR compared to untreated control cells. Pretreatment with the PKA inhibitor H7 (50 μmol/L) reduced but did not abrogate linaclotide‐induced apical CFTR traffic. In contrast, pretreatment with the PKG inhibitor H8 (70 μmol/L) reduced linaclotide‐induced apical CFTR traffic more potently compared to the PKA inhibitor H7. These results are consistent with a prominent role for PKGII signaling pathway in linaclotide‐induced trafficking of CFTR into the apical plasma membrane, but may indicate a role for cross‐activation through the PKA signaling pathway.

### Inhibition of PKGII attenuates linaclotide‐evoked transepithelial ion current in rat proximal colon

In rat intestine, expression of both PKGII mRNA and protein was shown in the small intestine, cecum, and proximal colon (Markert et al. 1995). In the rat proximal colon PKGII is predominantly localized in the apical membrane of crypts and surface epithelium (Markert et al. 1995). In order to evaluate the effect of PKGII inhibition on the linaclotide‐induced transepithelial ion current we used the PKGII inhibitor H8 (Golin‐Bisello et al. 2005). Pretreatment of rat proximal colonic epithelium with H8 (100 μmol/L) significantly decreased linaclotide‐induced I_sc_ compared to vehicle‐treated tissues (Fig. 7C). To confirm that H8 treatment had no effect on PKA‐mediated I_sc_, tissues were stimulated with a water‐soluble analog of the cAMP agonist forskolin, NKH477. Activation of PKA with NKH477 induced a robust transepithelial ion current, while preincubation of rat colonic mucosa with the PKGII inhibitor H8 had no effect on NKH477‐evoked I_sc_ suggesting that treatment with H8 has no effect on the PKA‐mediated short circuit current (Fig. 7C).

### Summary

**Figure 8:**
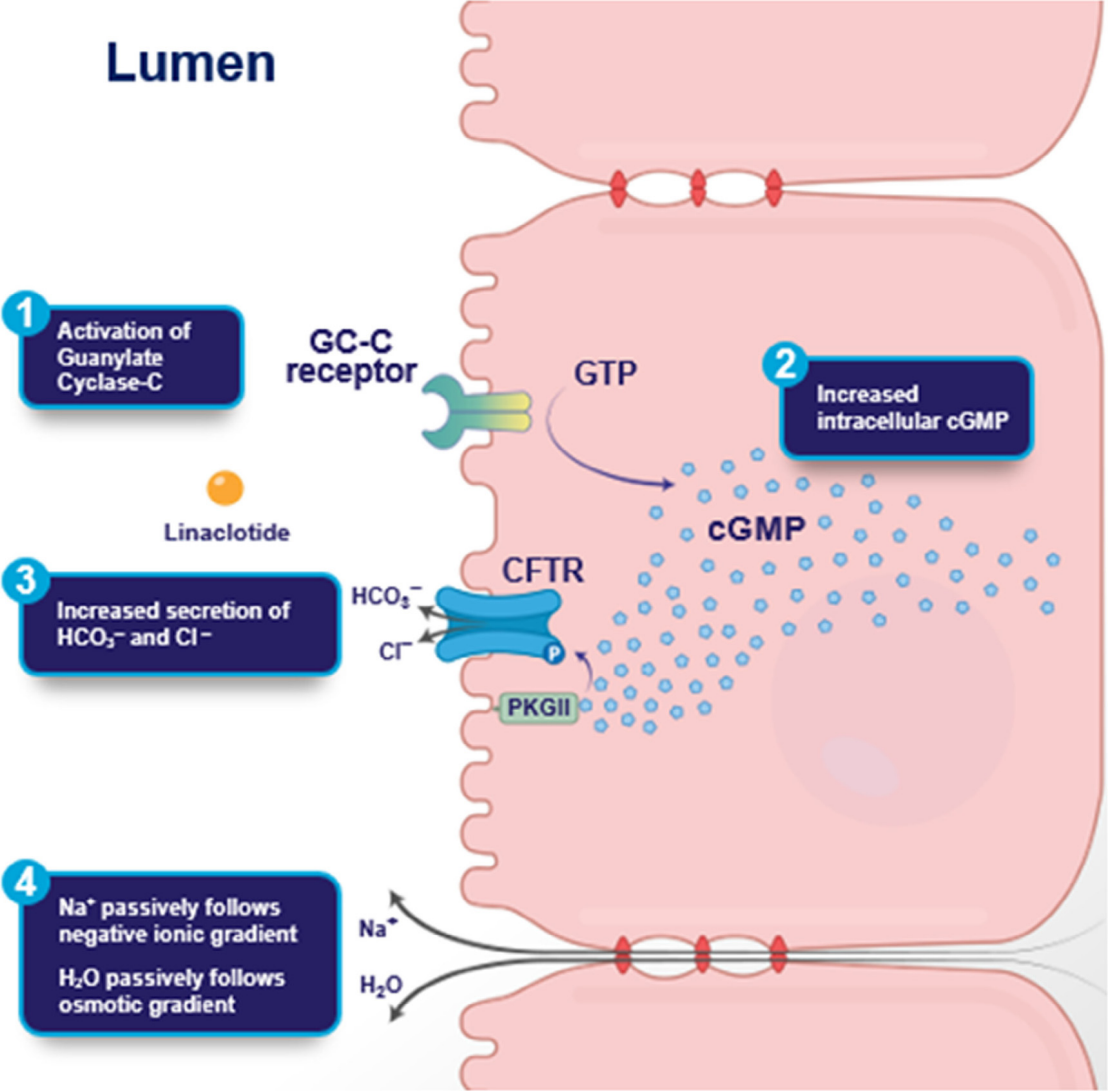
Schematic diagram of mechanism of action of linaclotide‐mediated signaling pathway. Proposed mechanism of action of the guanylate cyclase‐C (GC‐C) agonist linaclotide, through activation of the GC‐C/cyclic guanosine‐3′,5′‐monophosphate (cGMP) pathway, stimulating fluid secretion in the intestinal lumen. (1) Linaclotide binds to and activates GC‐C, expressed on the apical surface of intestinal epithelial cells. (2) Activation of GC‐C results in hydrolysis of guanosine triphosphate (GTP) and increased production of intracellular cGMP. (3) Increased cGMP concentrations activate cGMP‐dependent protein kinase, type 2 (PKG‐II), leading to phosphorylation and activation of the cystic fibrosis transmembrane conductance regulator (CFTR) ion channel, stimulating the secretion of chloride (Cl^−^) and bicarbonate (HCO3^−^) ions in the lumen. (4) CFTR‐mediated secretion of Cl^−^ and HCO3^−^ ions is accompanied by the passive efflux of sodium (Na^+^) ions and water (H_2_O), following the electronegative osmotic gradient. Due to its minor role in linaclotide‐stimulated GC‐C/cGMP‐dependent fluid secretion, PKA cross‐activation is not shown.

The main findings of this study elucidating the mechanism of action of linaclotide‐mediated fluid secretion in the intestine are illustrated in Figure 8. Linaclotide binding to GCC expressed on the apical brush border membrane of enterocytes activates the receptor, leading to increased levels of intracellular cGMP, which in turn activates cGKII. cGKII‐dependent phosphorylation activates the CFTR anion channel, stimulating increased CFTR‐dependent chloride and bicarbonate secretion into the intestinal lumen. The active transcellular movement of chloride and bicarbonate ions is accompanied by passive electroneutral paracellular movement of sodium ions and water efflux into the lumen. In rat jejunum and human colonic C2BBe and T84 cells, cGKII is expressed, and linaclotide stimulation of the GC‐C/cGMP/cGKII pathway further results in exocytic trafficking of CFTR from subapical vesicles into the membrane via this pathway. While the GC‐C/cGMP/PKGII pathway is the predominant pathway, it appears that in C2BBe and T84 cells, linaclotide stimulated cross‐activation of PKA may also be involved in CFTR trafficking.

## Discussion

Linaclotide is an FDA‐approved drug for the treatment of adult patients with Irritable Bowel Syndrome with Constipation (IBS‐C) and Chronic Idiopathic Constipation (CIC). As a potent agonist of GC‐C located on the luminal surface of the intestine, linaclotide activates CFTR‐dependent fluid secretion via a pathway initiated by increased conversion of guanosine triphosphate to cGMP (Busby et al. 2013). In the intestine, cGMP agonists stimulate fluid secretion by inducing phosphorylation of the CFTR anion channel (Vaandrager et al. 1997). However, the effect of linaclotide stimulating increased fluid secretion via a mechanism involving the activation of CFTR trafficking in the intestine was unknown. This study employed rat small intestine and two independent human colon‐derived intestinal cell models to examine the mechanisms of linaclotide signaling and fluid secretion involving trafficking of CFTR in the intestine. The data reveal that linaclotide elicits its fluid secretory effect in the intestine by activating the GC‐C/cGMP/PKGII and potentially PKA pathways, stimulating trafficking of CFTR to the intestinal cell surface. In the proximal small intestine, linaclotide elicits fluid secretion primarily through cGKII‐dependent trafficking of CFTR, while in the colon where cGKII machinery is less abundant, cross‐activation of PKA‐CFTR can occur.

Thus, we first examined linaclotide's ability to elicit fluid secretion and CFTR traffic into the surface of enterocytes in the rat jejunum because (1) this segment is the major site of CFTR expression in the intestine and fluid secretion, and (2) this segment exhibits the highest levels of cGKII. We found that linaclotide elicited a robust increase in fluid accumulation in jejunum loops similarly to the GC‐C agonist, STa enterotoxin (Golin‐Bisello et al. 2005), and as reported in human and mouse small intestine enteroids (Pattison et al. 2016). Linaclotide‐induced fluid accumulation was accompanied by a robust increase in surface CFTR when compared to the untreated intestine. Although saline treatment is often used as a control, this study examined surface CFTR in untreated, saline‐treated, and linaclotide‐treated jejunum because we previously observed saline (pH 7.4) induced trafficking of CFTR in rat intestine (Jakab et al. 2012). In our previous study, we examined the lumen pH in untreated rat intestinal segments and found that the mean baseline lumen pH in rat jejunum was 6.67. When rat jejunum loops were treated with saline pH 7.4, CFTR and other transporters were upregulated on the membrane, consistent with trafficking. Our interpretation of those observations was that the change in lumen pH leads to trafficking of membrane transporters, as we observed trafficking of transporters in response to pH changes from baseline in rat intestinal segments. Linaclotide treatment produced a tense fluid filled jejunum loop and the highest levels of surface CFTR. Consistent with our previous observations, saline treatment (pH 7.4) also increased surface CFTR levels relative to the untreated intestine, but was not associated with significant fluid accumulation (Jakab et al. 2012). Previously we demonstrated that in rat colonic epithelium linaclotide induces transepithelial ion current with EC_50_ = 9.2 nmol/L (Tchernychev et al. 2015). We used CFTR inhibitor GlyH‐101 to study CFTR‐mediated ion current evoked by low (6 nmol/L) and high (100 nmol/L) concentrations of linaclotide. Treatment with GlyH‐101 significantly inhibited Isc induced by low concentration of linaclotide. In contrast, inhibitory effect of GlyH‐101 was less profound when 100 nmol/L of linaclotide was used to stimulate rat colonic mucosa. This result implies that in rat proximal colon other anion transporters or channels might be activated in response to high concentration of linaclotide.

As cGKII is expressed in rat proximal colon, we used this segment further to examine whether linaclotide‐evoked current is mediated by activation of apical CFTR. The findings that linaclotide‐induced CFTR currents are consistent with the cGKII‐mediated activation and traffic of CFTR as observed in the colonic cell lines and rat colon as CFTR is regulated by second messenger traffic in all segments of the intestine (Jakab et al. 2011, 2012).

T84 cells are derived from a colorectal carcinoma and more closely resemble the colon crypt, while C2BBe, a clone derived from CaCo‐2 cells, is similarly derived from a colorectal adenocarcinoma but more closely resemble surface cells of the small intestine. In rat intestine, mRNA levels of cGKII were present in the small intestine and proximal colon but notably absent in distal colon, while highest levels of protein were observed in villus cells of the jejunum (Markert et al. 1995). Furthermore, T84 cells express cGKII mRNA but protein expression has not been confirmed (Selvaraj et al. 2000). We found detectable levels of cGKII mRNA and protein expression in both C2BBe and T84 cells, although expression was higher for either in the C2BBe cells. The higher level of cGKII expression in C2BBe is consistent with a more differentiated mature villus like small intestinal enterocyte phenotype for this cell model. In the absence of data on cGKII expression in human colon our data suggest that GC‐C agonists including linaclotide can utilize the cGKII pathway in colonic cell lines and the human distal colon. As phosphorylation of VASP at Ser^239^ reflects activation of cGKII (Oelze et al. 2000; Lawrence and Pryzwansky 2001) the observation that linaclotide elicited more robust phosphorylation of VASP^Ser239^ compared to 8Br‐cGMP in both C2BBe and T84 cells further support a prominent role for linaclotide in cGKII activation, a scenario that appears to distinguish cGKII pathways in rat and human distal colon. Our previous reported observation that linaclotide induces phosphorylation of VASP^Ser239^ was validated by others (Ahsan et al. 2015; Pattison et al. 2016).

We studied linaclotide potency in T84 and C2BBe cells using cGMP as a biomarker. Linaclotide induced cGMP synthesis with similar potency in both cell lines at concentrations of 1 μmol/L and 3 μmol/L, which approximately correspond to the EC_80_ and EC_100_ of linaclotide in these cells.

Linaclotide stimulation of intestinal epithelium derived from different segments of the human intestine resulted in increased levels of both intracellular and extracellular cGMP. While the increase in intracellular cGMP following ligand activation of GC‐C is well established, the efflux of cGMP from enterocytes is not well studied. Recently we demonstrated that linaclotide and the natural GC‐C agonist uroguanylin induce cGMP efflux from the apical and basolateral membrane of mouse and rat colonic mucosa (Silos‐Santiago et al. 2013; Tchernychev et al. 2015) as well as from the human colonic Caco‐2 cell line (Castro et al. 2013; Silos‐Santiago et al. 2013). This cGMP efflux is probenecid sensitive (Castro et al. 2013; Silos‐Santiago et al. 2013) and in part mediated by apical MRP4 (Tchernychev et al. 2015) and some yet unidentified transporter(s) located on apical and basolateral membranes. While linaclotide‐mediated elevation in intracellular cGMP promotes electrolyte secretion and intestinal transit (Bryant et al. 2010; Busby et al. 2010), increased levels of extracellular cGMP secretion into the submucosa following linaclotide stimulation mediate antinociceptive effects in several models of colonic hypersensitivity (Castro et al. 2013; Silos‐Santiago et al. 2013; Hannig et al. 2014).

Based on the expression of cGKII in C2BBe and T84 cells, combined with the observed potent linaclotide‐induced phosphorylation of VASP at residue Ser^239^, we predicted that linaclotide would stimulate CFTR traffic into the brush border membrane, similarly to the GC‐C agonist STa (Golin‐Bisello et al. 2005). The higher concentration of linaclotide resulted in higher levels of VASP^Ser239^ phosphorylation compared to the lower concentration in both C2BBe and T84 cells. However, this higher concentration resulted in higher surface CFTR levels in C2BBe cells compared to T84 cells. This finding is consistent with the higher levels of cGKII expression in C2BBe cells compared to T84 cells, and the observations that highest levels of cGKII expression are found in villus enterocytes of the small intestine.

Because cAMP and PKA are potent regulators of CFTR traffic from subapical endosomes into the brush border membrane in the intestine, and the cGMP and cAMP pathways can converge in the colon, the contribution of the cGKII pathway to linaclotide‐induced traffic of CFTR was examined by silencing cGKII expression. Cells lacking cGKII expression (~50%) only marginally recruited CFTR to the membrane following linaclotide stimulation, but were able to recruit CFTR to the membrane following addition of the cAMP agonist dibuturyl cAMP, confirming the important role for cGKII in linaclotide‐induced traffic of CFTR. Examination of linaclotide‐induced traffic of CFTR in the presence of pharmacologic inhibition of PKA (H7) or PKGII (H8) in cGKII‐KD cells confirmed some cross‐activation of the cGKII‐PKA pathway in linaclotide‐induced traffic of CFTR in both cell lines as the PKA inhibitor partially abrogated linaclotide‐stimulated traffic. These studies suggest that in the human colon, linaclotide‐induced traffic of CFTR primarily employs the cGKII pathway, but also to a minor degree cross‐activates the PKA pathway.

Activation of PKGII signaling pathway in rat proximal colon by linaclotide was confirmed using the PKGII inhibitor H8. Treatment of rat proximal colonic epithelium with H8 inhibited linaclotide‐evoked I_sc_ (Fig. 6C), consistent with the expression of cGKII in this segment. In contrast, H8 had no effect on PKA‐mediated short circuit current induced by NKH477 (Fig. 6C). While inhibition of linaclotide‐induced I_sc_ by H8 was significant, it was not complete suggesting activation of a PKGII‐independent signaling pathway. Similarly, significant but incomplete inhibition of GC‐C‐mediated transepithelial ion current in rat proximal colon by PKGII antagonists H8 and Rp‐8‐pCPT‐cGMPS has been reported previously (Vaandrager et al. 1997). Furthermore, only minor attenuation of STa‐induced transepithelial ion current was observed in the proximal colon, but not in the jejunum of PKGII‐deficient mice (Vaandrager et al. 2000). One of the well‐characterized intracellular targets of cGMP is PDE3 which belongs to the family of cGMP‐inhibited cyclic nucleotide phosphodiesterases (Manganiello et al. 1995). The PDE3 inhibitor amrinone induced transepithelial ion current in tissues from normal and PKGII knockout mice confirming the presence of this enzyme in colonocytes (Vaandrager et al. 2000), indicating that cGMP may act by inhibiting PDE3 which results in a local cAMP accumulation and activation of the PKA pathway (Vaandrager et al. 2000). This cGMP‐PDE3‐dependent cross‐activation of PKA pathway may help explain the incomplete inhibition of linaclotide‐induced Isc by compound H8. Furthermore, in human ascending colon, the linaclotide‐mediated increase in intracellular cGMP evoked a transepithelial ion current, which was sensitive to the NKCC1 inhibitor bumetanide, suggesting that this current is mediated by transcellular flux of Cl^−^ anions. In this regard, our results are consistent with previously published data describing the effect of guanylin and STa on electrogenic anion transport in intestinal epithelium isolated from human ascending colon (Kuhn et al. 1994).

In summary, the findings presented in this study provide further mechanistic insights into the effects of linaclotide, specifically its ability to stimulate markedly increased fluid secretion in the small and large intestine by upregulating the amount of cell surface CFTR via the GC‐C/cGMP/cGKII‐dependent pathway. This mechanism of linaclotide is likely involved in the improvement of bowel symptoms in adult IBS‐C and CIC patients treated with linaclotide.

## Conflict of Interest

No conflicts of interest, financial, or otherwise are declared by the author(s).
